# A Practical Method to Estimate the Resolving Power of a Chemical Sensor Array: Application to Feature Selection

**DOI:** 10.3389/fchem.2018.00209

**Published:** 2018-06-12

**Authors:** Luis Fernandez, Jia Yan, Jordi Fonollosa, Javier Burgués, Agustin Gutierrez, Santiago Marco

**Affiliations:** ^1^Department of Electronics and Biomedical Engineering, Universitat de Barcelona, Barcelona, Spain; ^2^Signal and information processing for sensing systems, Institute for Bioengineering of Catalonia, Barcelona, Spain; ^3^College of Electronic and Information Engineering, Southwest University, Chongqing, China; ^4^Departament d'Enginyeria de Sistemes, Automàtica i Informàtica Industrial (ESAII), Universitat Politecnica de Catalunya, Barcelona, Spain; ^5^Institut de Recerca Pediàtrica Hospital Sant Joan de Déu, Barcelona, Spain

**Keywords:** gas sensor array, MOX sensors, Resolving Power, sensor resolution, dimensionality reduction, machine olfaction

## Abstract

A methodology to calculate analytical figures of merit is not well established for detection systems that are based on sensor arrays with low sensor selectivity. In this work, we present a practical approach to estimate the Resolving Power of a sensory system, considering non-linear sensors and heteroscedastic sensor noise. We use the definition introduced by Shannon in the field of communication theory to quantify the number of symbols in a noisy environment, and its version adapted by Gardner and Barlett for chemical sensor systems. Our method combines dimensionality reduction and the use of algorithms to compute the convex hull of the empirical data to estimate the data volume in the sensor response space. We validate our methodology with synthetic data and with actual data captured with temperature-modulated MOX gas sensors. Unlike other methodologies, our method does not require the intrinsic dimensionality of the sensor response to be smaller than the dimensionality of the input space. Moreover, our method circumvents the problem to obtain the sensitivity matrix, which usually is not known. Hence, our method is able to successfully compute the Resolving Power of actual chemical sensor arrays. We provide a relevant figure of merit, and a methodology to calculate it, that was missing in the literature to benchmark broad-response gas sensor arrays.

## Introduction

Analytical figures of merit are well understood for mature chemical instrumentation (Olivieri, 2014). For univariate zero order chemical sensors, figures of merit can be estimated as well using conventional recommendations from IUPAC (Justino et al., 2010). However, the computation of figures of merit is not clear in many scenarios in which researchers and practitioners prefer to address the problems using arrays of solid-state chemical sensors, particularly in gas phase. Solid state sensors usually do not have peak shape responses as it may happen in chromatography, spectrometries, and spectroscopies. These sensors are typically characterized by very poor selectivity, non-linearities, and sensor instabilities (Hierlemann and Gutierrez-Osuna, 2008). Similarly, conventional analytical instrumentation show degraded performance when miniaturized using microsystems technologies. Examples are the integration of Non-Dispersive Infrared Sensors with overlapping sensitivities across the spectral domain (Calaza et al., 2003; Rubio et al., 2006, 2007; Fonollosa et al., 2009) the integration of mass spectrometers in MEMS technologies (Syms and Wright, 2016) or the integration of miniature Gas Chromatographers (Zampolli et al., 2009). Figures of merit also need reconsideration for direct-sampling fast-analysis techniques, such as Ion Mobility Spectrometry (Borsdorf and Eiceman, 2006) or Direct Analysis Real Time-Mass Spectrometry (Gross, 2014). In many of these instrumental configurations, the interest is not targeted selective detection of certain analytes, but global fingerprint analysis using chemometrics (Pavlovich et al., 2016; Szymanska et al., 2016).

The definition of figures of merit for chemical sensor arrays was first considered in the pioneering work of Davide et al. (1993) and later by Snopok (Snopok and Kruglenko, 2002). In most occasions, figures of merit for chemical sensor arrays have been proposed in the context of sensor array design (Johnson and Rose-Pehrsson, 2015). The direct translation of tensorial figures of merit to sensor systems has been used by Marth (Marth et al., 1999). However, most classical definitions assume linearity of response and this limits their applicability in non-linear sensors. Recently, Burgués et al. studied the application of the Limit of Detection (LOD) definitions by IUPAC to metal oxide sensors (MOX), since most of the underlying statistical hypothesis of the theoretical development may not hold for chemical sensors (Burgués and Marco, 2017; Burgués et al., 2018). Also concerning LOD, Fonollosa et al. propose an information theory approach to weigh the uncertainty of the sensor response with the prior knowledge about the probability of analyte presence in the sample (Fonollosa et al., 2014). Similarly, Johnson and Knapp have proposed alternative definitions of selectivity based on the Cramer-Rao lower bounds (Johnson and Knapp, 2017).

Resolving Power (RP) and *Resolution* (R) are two key figures of merit in chemical instrumentation. However, there is some confusion between these two terms, and sometimes they are mixed up (Cohen et al., 2008). IUPAC has done, over the years, a tremendous effort to clarify terminology in chemical sciences.

In analytical chemistry theory, Resolving Power measures are usually defined for peak-shaped signals. Nevertheless, in sensor arrays, and due to the poor sensor selectivity, analyte discrimination is often based on the use of pattern recognition algorithms, as already acknowledged in the classic definition of electronic nose introduced by Gardner (Gardner and Bartlett, 1994). For the interested reader, the use of pattern recognition algorithms for chemical sensor arrays has been reviewed by Gutierrez-Osuna (2002) and Marco (Marco and Gutierrez-Galvez, 2012).

Today, the combination of analytical instrumentation and sensor systems for the identification of complex chemical objects using pattern recognition and machine learning techniques is widespread. One can find hundreds of examples in the literature, but just for illustration purposes, we will mention only a few. Kuske et al. used chemical sensor arrays to discriminate mold species growing on building materials (Kuske et al., 2006). Garrido-Delgado et al. used Ion Mobility Spectrometry to classify wines according to their Certified Brand of Origin (CBO) (Garrido-Delgado et al., 2011). Cauchi et al. used Gas Chromatography Data in combination with Partial Least Squares-Discriminant Analysis (PLS-DA) for the diagnosis of diverse gastrointestinal diseases using various body samples (Cauchi et al., 2014). Vaclavik et al. have used Direct Analysis in Real Time Mass Spectrometry (DART-MS) in combination with Fisher Discriminant Analysis for authenticity assessment in olive oil samples (Vaclavik et al., 2009).

Over the years, heuristic measures of the Resolving Power of chemical sensor arrays have been proposed. In most cases, they are versions of the Fisher score that compute a ratio between the mean distance between classes and the mean dispersion of the classes (Doleman et al., 1998; Muezzinoglu et al., 2010; Xu et al., 2010; Vergara and Llobet, 2012; Magna et al., 2018).

From a formal point of view, the discrimination of classes in a multidimensional space is similar to the detection of symbols in digital communication theory. Claude Shannon, in his mathematical theory of communication in the presence of noise (Shannon, 1984), posed the problem of the number of signals (or symbols) that can be distinguished by the receiver despite the presence of noise. He proposed a ratio between the power of the signal plus noise and the power of the noise in a multidimensional setting.

Along the same lines, Gardner and Bartlett (1996) introduced the “*range*” as a ratio between the signal span in the input feature space and the noise hyper-volume. This concept was further developed by Pearce et al. (Pearce, 2000; Pearce and Sánchez-Montañés, 2004) and they proposed the means to calculate the hyper-volume of the signal span based on the sensor sensitivity matrix for linear sensors and the integration of the Jacobian matrix for non-linear sensors.

However, years later, no practical applications of the method can be found in the literature. We believe that this particular situation is caused by three reasons: First, their method experiences a notable increment of complexity when it is applied to arrays of non-linear sensors and/or to arrays subjected to heteroscedastic sensor noise. Second, the application of the technique is limited to discrimination tasks where the amount of sensor features does not exceed the number of pure gases. This restriction constitutes a severe shortcoming since current sensor arrays tend to provide large amounts of data per sample (LaFratta and Walt, 2008; Beccherelli et al., 2010; Marco et al., 2014). Third, it is not easy to obtain the sensitivity matrix between chemical stimuli and the sensor responses, especially for non-linear sensors. The sensitivity matrix is then used to compute the hyper-volume of the sensor space.

In this work, we present a practical approach to the estimation of the Resolving Power as defined first by Shannon and for chemical sensor arrays by Gardner and Barlett. Our method combines dimensionality reduction and proposes the use of algorithms to compute the convex hull of the empirical data to estimate the signal volume in the input feature space. We explore this concept first with synthetic data and then with empirical data from temperature modulated MOX sensors. We explore how this concept can be practically applied to mild non-linear sensors by the partition of the input feature space.

## Resolving power and resolution in chemical sensor arrays

IUPAC definitions of Resolving Power and resolution are linked to certain analytical techniques. For instance, in mass spectrometry and for a single peak made up of singly charged ions at mass *m* in a mass spectrum, the resolution may be expressed as

(1) $$\begin{array}{l} \frac{\Delta m}{m} \end{array}$$

where Δ*m* is usually the full width half maximum (FWHM) and *m* is the mass center of the peak (Todd, 1991). On the other hand, the IUPAC definition of Resolving Power is: “*For* two *peaks of equal height with masses m*_1_ *and m*_2_ *when there is overlap between the* two *peaks to a stated percentage of either peak (10% is recommended), then the* Resolving Power *is defined as m*_1_*/(m*_1_*-m*_2_*)*” (Nič, 1997). Note that a smaller resolution means an enhanced figure of merit, while the contrary holds for the Resolving Power.

Instead, for chromatography, IUPAC uses the term *peak resolution* with a meaning closer to the concept of Resolving Power. In particular, peak resolution is defined in chromatography as a characteristic separation of two adjacent peaks.

(2) $$\begin{array}{l} R_{AB}=2\frac{\left|t_{A}-t_{B}\right|}{\left|w_{A}+w_{B}\right|} \end{array}$$

where *R*_*AB*_ is peak resolution and *t*_*A*_ and *t*_*B*_ are the retention times for compounds A and B and *w*_*A*_ and *w*_*B*_ are the widths of the peaks at the base (Nič, 1997).

In optical spectroscopies, according to the IUPAC, the Resolving Power is the transition wavenumber (or wavelength or frequency) divided by the resolution. The transition wavenumber is the difference between two energy states and the resolution is defined as the minimum wavenumber, wavelength or frequency difference between two lines in a spectrum that can be distinguished (Nič, 1997). We can notice that in mass spectrometry resolution is a dimensionless quantity, but the definition of resolution for optical spectroscopies has physical units (wavelength, wavenumber, or frequency).

For ion mobility spectrometry, Rokushika et al. (1985) define resolution as:

(3) $$\begin{array}{l} R=\frac{t}{2W} \end{array}$$

where *t* is the drift time of the ion pulse and W is the width of the ion pulse at FWHM.

On the other hand, we have to consider as well, that the term *resolution* is also used in Metrology and Measurement Science as the “smallest quantity being measured that causes a perceptible change in the corresponding indication.” See, for instance, the definition issued by the Bureau International des Poids et Measures (BIPM) in the document “International vocabulary of metrology: basic and general concepts and associated terms” (Joint committee for Guides in Metrology, 2012). This definition is commonly accepted in sensor science, where the perceptible change is related to the noise level in the sensor output. For instance, for univariate sensors, resolution is defined by D'Amico (D'Amico and Di Natale, 2001) as:

(4) $$\begin{array}{l} Res\left(x\right)=\underset{V_{out}\to V_{n}}{\text{lim}}\frac{V_{out}\left(x\right)}{S\left(x\right)} \end{array}$$

where *Res(x)* is the resolution, *V*_*out*_*(x)* is the corresponding sensor output and *S(x)* the sensitivity at the working point *x*, being *x* the sensor input. *V*_*n*_ is the noise level and it is taken as *k*σ, where σ is the standard deviation of the noise and *k* is a multiplicative factor. In sensor science there is no consensus on the value of *k*, though common values are *k* = *1, k* = *3*, or even *k* = *10*. With these considerations in mind, when reporting resolution, it is important to state the working point for non-linear sensors and the used value of *k*. At this point it is easy to link the evaluation of resolution at the blank level with the definitions of the limit of detection (LOD) and limit of quantification (LOQ) in analytical chemistry (Olivieri, 2014; Desimoni and Brunetti, 2015; Burgués et al., 2018).

In this context, a related concept is the signal-to-noise ratio. Faber et al. have proposed a definition for signal-to-noise ratio in a tensorial framework (Faber et al., 1997). For first-order calibration they define the signal to noise ratio as:

(5) $$\begin{array}{l} \frac{S}{N}=\frac{r_{i}^{NAS}}{\sigma \left(r_{i}^{NAS}\right)} \end{array}$$

where the system response for analyte *i* is taken as the Net Analyte Signal (NAS) (Bro and Andersen, 2003; Ferré and Faber, 2003), and the noise is evaluated as the standard deviation of the measurement noise in the NAS projection. Usually, the NAS is taken to be a unidimensional vector.

In occasions, instead of the signal-to-noise ratio, the sensor community prefers to use the dynamic range (*DR*), defined as the maximum concentration with respect to the LOD:

(6) $$\begin{array}{l} DR=\frac{\max\left(C\right)}{LOD}=\;\frac{Range}{LOD} \end{array}$$

where *Range* is the maximum concentration *C* at which the detector is still sensitive. In other occasions, researchers prefer the term linear dynamic range, and then the maximum concentration refers to the maximum concentration of the analyte that keeps the calibration curve linear.

In this context, a different approach to the estimation of the joint discrimination power and resolution is needed. This definition should be established in the input space taking into account the span of the feature vectors and the noise intensity for each cluster.

From a formal point of view, the discrimination of classes in a multidimensional space is similar to the detection of symbols in digital communication theory. Claude Shannon, in his mathematical theory of communication in the presence of noise (Shannon, 1984), posed the problem of the number of signals (or symbols) that can be distinguished by the receiver despite the presence of noise. He proposed that this number could be estimated as follows. If the signal has a power *S* and the noise has a power *N*, the number of signals that can be well-distinguished is:

(7) $$\begin{array}{l} K\sqrt{\frac{S+N}{N}} \end{array}$$

where the noise is assumed to be additive. *K* is a constant close to 1 that depends on the allowed error to separate the different signals.

### Gardner and barlett proposal

Gardner and Bartlett (1996) introduced a concept that they named *range* defined as the maximum number of input conditions that can be discriminated in sensor space in the presence of noise:

(8) $$\begin{array}{l} N_{n}=\frac{\prod_{i\;=\;1}^{n}FSD\left(S_{i}\right)}{V_{n}} \end{array}$$

From our point of view, this definition can be better described by the term “Resolving Power,” and this is how from now on, we will refer to this definition. In order to avoid confusion, we will use the term “range” only to refer to the maximum concentration at which the sensor remains sensitive.

Here, the sensor space is a space spanned by the sensor signals, *n* is the number of sensors,

(9) $$\begin{array}{l} V_{s}=\overset{n}{\underset{i\;=\;1}{\prod }}FSD\left(S_{i}\right) \end{array}$$

is the full-scale deflection of the output *S*_*i*_ of the *i-th* sensor, and *V*_*n*_ is the hyper-volume of noise in the sensor space and is defined by Equation (10):

(10) $$\begin{array}{l} V_{n}=\frac{2\pi^{\frac{n}{2}}\prod_{i\;=\;1}^{n}\sigma_{i}}{n\Gamma \left(\frac{n}{2}\right)} \end{array}$$

where the σ_*i*_ is the standard deviation of noise for *i*-*th* sensor and Γ is the Gamma function. It is worth noticing that *V*_*n*_ is a function of the sensor output and thus is not in general uniform over the whole sensor space. Gardner and Barlett just introduced the theoretical framework to quantify the ability of a chemical sensor array to discriminate sensor stimuli but did not present a methodology that can implement it. In this work, the Resolving Power of a sensor array is estimated adapting the computation of the figure of merit in case of: (a) large sensor arrays, (b) non-linear sensor, (c) heteroscedastic sensor noise.

### Estimation of the hyper-volume of sensor space

According to Pearce (2000) in order to obtain the hyper-volume of sensor space, a simple linear analytical model was used to model the mapping relationship between *n* such sensors, each with potentially different sensitivity terms *s*_*ij*_ (*i* = 1, 2,…, *n*; *j* = 1, 2,…, *m*), and *m* stimuli. That means the stimuli space and sensor space are related through a space transformation dictated by the sensitivity matrix ***S***. Here, the *stimuli space* is defined as all the combinations of chemical stimuli that are possible. It must be remarked that such transformation is local if non-linearities are present in the sensors' responses. However, for the simplified case of linear sensors, ***S*** is a matrix of constant coefficients and the transformation becomes global:

(11) $$\begin{array}{l} X=SY \end{array}$$

where ***X*** is the response of sensors (sensor space) and ***Y*** would be defined as concentrations to every single chemical source (stimuli space). From empirical data ***S*** may be estimated by classical least squares if one knows all the constituents of the sample.

The element *x*_*ki*_ represents, the response of the *i-th* sensor to the *k-th* stimuli. Note that Pearce and Sánchez-Montañés (2004) considered only sensor arrays in which each of the individual sensors provided a single feature. This notation is not constrained to any particular type of sensor or number of elements in the sensor array. It can be extended to the case in which sensors operate under some parameter modulation (e.g., temperature in MOX sensors), since each of the working conditions can be considered as a virtual sensor in the array, and in turn, a new feature to the sensor space (or, in other words, a new column in the matrix ***X***).

The hyper-volume spanned by the gas mixtures in the stimuli space (*V*_*o*_) can be projected onto the sensor space, giving rise to the *hyper-volume of sensor space* (*V*_*s*_). In Pearce work (Pearce, 2000), the computation of *V*_*s*_ is straightforward for linear sensors. If ***S*** is a square matrix, namely if the number of pure chemical sources equals to the number of features, *V*_*s*_ is computed as:

(12) $$\begin{array}{l} V_{s}=V_{o}|\det\left(S\right)| \end{array}$$

The reader is referred to Pearce (2000) for extended study of non-square matrices.

The method developed by Pearce to estimate *V*_*s*_ requires knowing the sensitivity matrix obtained by fitting a direct classical least squares (CLS) model that relates stimuli and sensor spaces to project *V*_*o*_ onto the sensor space (Equation 12). It is worth to mention that this information is not usually available. In most practical cases direct classical least squares models (CLS) are not fit, because the full set of compounds in the sample is unknown. Consequently, inverse calibration models are the preferred approach.

On the contrary, what we usually know is the set of sensor responses to a collection of mixtures, that is, the sensor space. Thus, a direct computation of *V*_*s*_ from the sensor space seems to be a reasonable alternative. One way to estimate *V*_*s*_ consists in computing the convex hull of the sensor space. The convex hull problem is one of the main issues in computational geometry. Computing the convex hull stands for creating a univocal effective representation of a convex shape. The computational cost of estimating the convex hull of a finite set of points depends on the parameters *n* and *h*, which are, respectively, the number of points to be enclosed and the number of points on the convex hull. In this sense, the worst case scenario for convex hull computation appears when the points are distributed on a hyper-surface, since *h* equals *n*. Convex hull algorithms can be used to estimate areas, volumes and hyper-volumes. Regarding the planar case, a number of algorithms to solve the convex hull problem have been developed, including: Gift wrapping -*O(nh)*, Graham scan -*O(n*log*n)*, Andrew's algorithm -*O(n*log*n)*, Divide and Conquer -*O(n*log*n)*, Chan's Algorithm -*O(n*log*n)*, and Quickhull -*O(n*log*n)*, among others (De Berg et al., 2000). For the case of spaces of dimension three of higher, the computation of volumes and hyper-volumes is generally performed using the Quickhull algorithm (Barber et al., 1996).

In this work, we compute the convex hull utilizing the Quickhull algorithm. Our choice for this algorithm is based on its ability to efficiently estimate hyper-volumes in two-dimensional spaces and in higher dimensionalities as well. Consequently, Quickhull algorithm allows computing *V*_*s*_ of sensor arrays comprising several sensor units, provided that sensor space contains data points properly sampled. In a nutshell, the Quickhull algorithm computes the convex hull in the following way: (1) First, it finds the *n* samples with the most extreme coordinates of the space (where *n* is the dimension of the space). (2) Next, it generates a hyper-plane using these *n* samples that halves the space into two subsets of samples. (3) After that, it seeks for the farthest sample with respect to the hyper-plane. The previous *n* samples along this one define a *facet*. (4) The samples within the *facet* are ignored by the algorithm in next steps because they do not belong to convex hull. (5) Steps (3) and (4) are repeated on the *edges* of the *facet*, with the exception of the initial hyper-plane. (6) The process continues until recursion termination and the selected samples generate the convex hull. The pseudo-code of the employed algorithm is shown in Table 1.

**Table 1:** Pseudo-code of the Quickhull algorithm, used to compute the hyper-volume.

**Algorithm 1** Estimate convex hull.
**1**: **procedure** QUICKHULL*(A)*
**2**: STEP 1) Find the *n* samples with the most extren1e coordinates of the space.
**3**: STEP 2) Define hyper-plane using these *n* samples that splits the space in two subsets of samples.
**4**: STEP 3) Find the farthest sample with respect to the hyper-plane. This sample together with the samples defining the hyper-plane form a *facet*.
**5**: STEP 4) Ignore the samples within the facet because they do not belong to convex hull.
**6**: STEP 5) Repeat steps 3) and 4) on the edges of the facet, with exception of the initial hyper-plane.
**7**: STEP 6) The process continues until recursion termination and the selected samples generate the convex hull.
**8**: **end procedure**

### Estimation of the hyper-volume of noise

**Figure 1:**
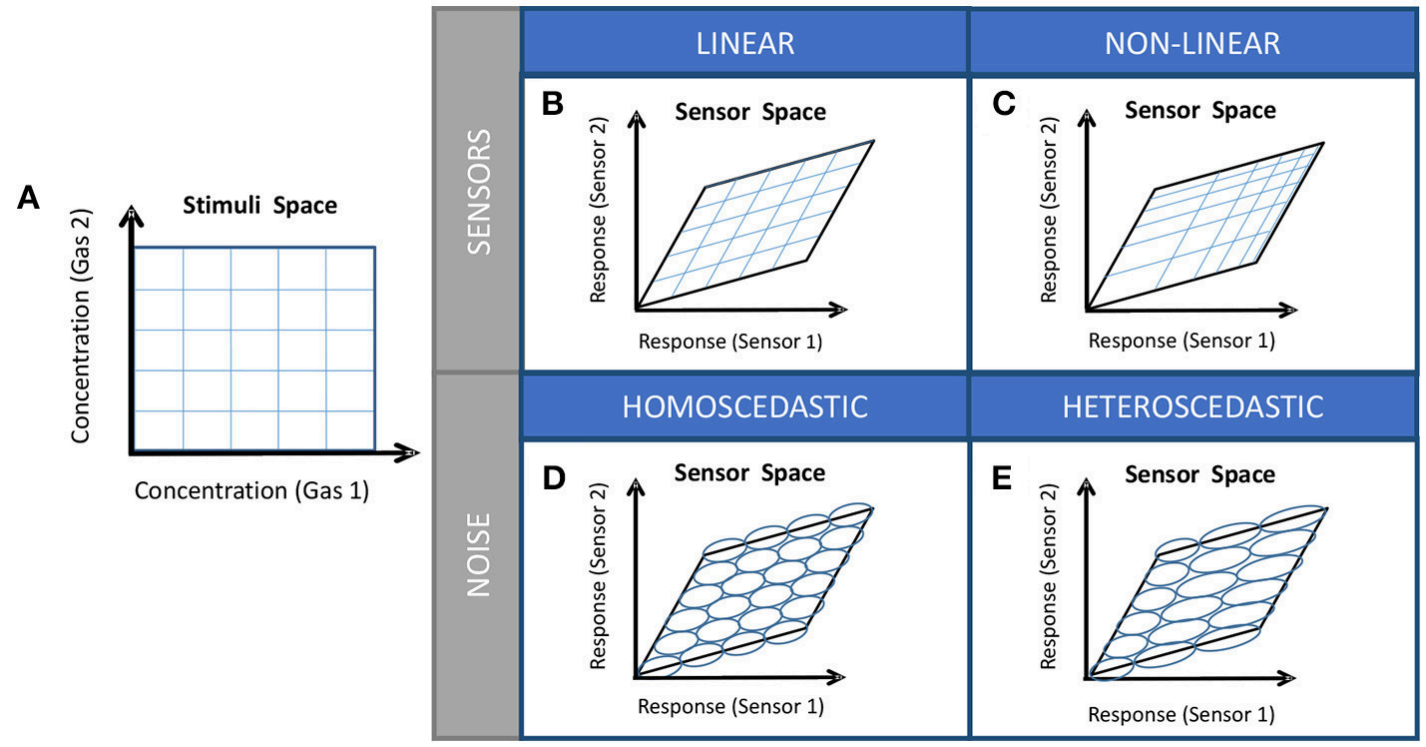
**(A)** Stimuli space, **(B)** Image of the stimuli space (sensor space) in case of partially selective linear sensors, **(C)** Image of the sensor space in case on partially selective non-linear sensors, **(D)** Sensor space with homoscedastic sensor noise, **(E)** Sensor space with heteroscedastic noise. The computation of the Resolving Power *N*_*n*_ is trivial only for the combination of cases **(B,D)**.

Theoretically, the value of *V*_*s*_ is computable in sensor spaces of any dimensionality, but it is unpractical for high-dimension spaces. This is because estimating *V*_*s*_ requires sampling the whole space, and the number of samples needed grows exponentially with the dimension of the space (Gutierrez-Osuna, 2002). The same applies to the estimation of *V*_*n*_. Consequently, it is preferable to reduce the dimensionality of the sensor space for obtaining better estimations of *V*_*s*_, *V*_*n*_, and *N*_*n*_. It is worth to mention that for the case of multi-sensor platforms, in which each sensor in the array follows a different transducing mechanism, sensor auto-scaling may be necessary before reducing the dimensionality of the sensor space. The dimensionality reduction of the sensor space can be conducted applying projection techniques such as Principal Component Analysis (PCA). Figure 1 shows the stimuli space and the corresponding responses of the sensors for different sensor and noise behaviors. In the case of linear sensor responses, the sensor space becomes an equally spaced grid. However, in the general case of non-linear responses, the grid becomes non-uniform for the same change of the input intensity. Similarly, for the case of homoscedastic noise, the dispersion caused by the noise effects is the same around any of the points in the sensor space. However, for the general case of heteroscedastic noise, the shape and size of the “cloud” change across the sensor space. Only, in the case of linear sensors under homoscedastic noise conditions, the estimation of the hyper-volume of noise is constant along the sensor space. For the general scenario of non-linear sensors, one needs another approach to estimate the hyper-volume of noise.

The estimation of the hyper-volume of noise *V*_*n*_ cannot in general be computed with Equation (10), because it, does not consider the correlation among sensors or features. In particular, assuming homoscedastic noise, we estimated the hyper-volume of noise from the square root of the determinant of the pooled covariance matrix.

### Proposed methodology to estimate the resolving power in a sensor array

**Figure 2:**
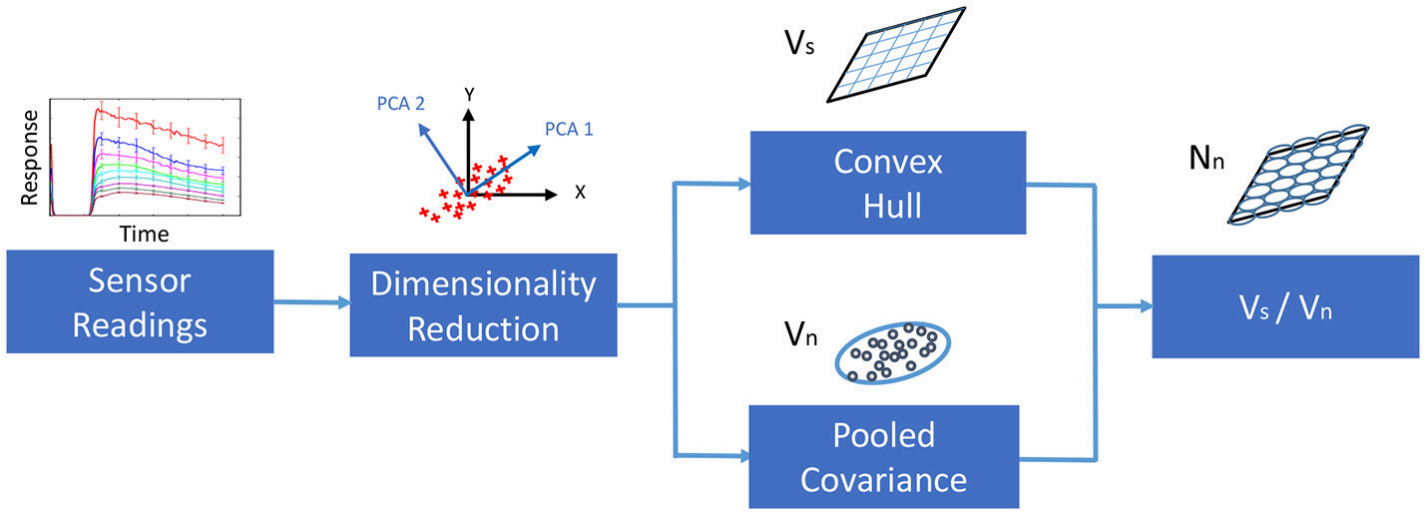
Workflow for estimating the Resolving Power of an array of sensors.

Hence, our approach to estimate the Resolving power of a sensor array begins with a dimensionality reduction using Principal Component Analysis. Then, the hyper-volume of sensor space, *V*_*s*_, is estimated from the convex hull that encloses the sensor responses in the new space. The pooled covariance is used to estimate the volume of noise, *V*_*n*_. Finally, the Resolving power, or the number of input stimuli that can be distinguished becomes the ratio of the hyper-volume of noise over the hyper-volume of the sensor space. Figure 2 summarizes the workflow to estimate the Resolving Power of a sensor array. It is worth to note that, in the case of non-linear sensors with heteroscedastic sensor noise, one can always split the region of interesting sub-regions, in which the Resolving Power can be estimated again. This partitioning of the region enables the estimation of the Resolving Power in non-linear scenarios.

## Materials and methods

### Synthetic datasets

We generated 2001 synthetic datasets to evaluate the impact of sensor similarity on the Resolving Power of an array of sensors. To simulate the response of an array of sensors to a gas mixture, we employed the Clifford-Tuma MOX sensor model (Clifford and Tuma, 1983) (Equation 13):

(13) $$\begin{array}{l} X_{i}={\left(1+\overset{n}{\underset{j\;=\;1}{\sum }}K_{j}C_{j}^{\alpha_{j}}\right)}^{-\beta } \end{array}$$

where *X*_*i*_ denotes the output resistance of the *i-th* sensor, *C*_*j*_ *(j* = *1, 2*, ···*, n)* denotes the concentration of the *j-th* compound of the gas mixture, *n* is the number of gases, *K*_*j*_ is the sensitivity coefficient of the sensor to the *j-th* compound, α_j_ is and integer or fractional power for the concentration of the *j-th* compound, and β is a non-integer exponent for the power law nature of the MOX sensors.

In our research, we limited for convenience both the number of sensors and the number of gases (*n*) to two, and we supposed that α_1_ = α_2_ = 1 and β = 0.5. Consequently, synthetic data was computed from the simplified sensor models *X*_1_ = (1 + *K*_11_*C*_1_ + *K*_12_*C*_2_)^−0.5^ and *X*_2_ = (1 + *K*_21_*C*_1_ + *K*_22_*C*_2_)^−0.5^. The range of concentration was the same for the two gases: *C*_j_ ε [100, 1,000] ppm. Note that, according to their models, both sensors exhibit the same response to one mixture when *K*_11_ = *K*_21_ and *K*_12_ = *K*_22_. Furthermore, the first and second sensors tend to be more selective, respectively, toward the first and second gases when |*K*_11_ – *K*_21_| >> 0 and |*K*_22_ – *K*_21_| >> 0. Hence, the Resolving Power of the array can be modified by tuning the sensibility parameters: *K*_11_, *K*_12_, *K*_21_, and *K*_22_.

We changed the sensitivities of both sensors using 2001 combinations of *K*_*ij*_, obtaining 2001 different sensor datasets. This was done by setting that the first sensor had a high sensitivity to the first gas and hardly any to the second in the beginning. Then, we decreased *K*_11_ and increased *K*_12_ according to the exponential rules *K*_11_*[k]* = 0.12^1+k·0.001^, and *K*_12_*[k]* = 0.12^5−k·0.001^, where *k* = 0, 1, 2, ···, 2,000. For the second sensor, we implemented the opposite changing rule from the first sensor. That is, the second sensor was selective to for the second gas at first, and then *K*_21_ and *K*_21_ increased and decreased, respectively, as *K*_11_ and *K*_12_. For *k* = 2,000, *K*_11_ = *K*_12_ = *K*_21_ = *K*_22_ = 0.12^3^, which means that both sensor present the same sensitivities for both gases.

**Figure 3:**
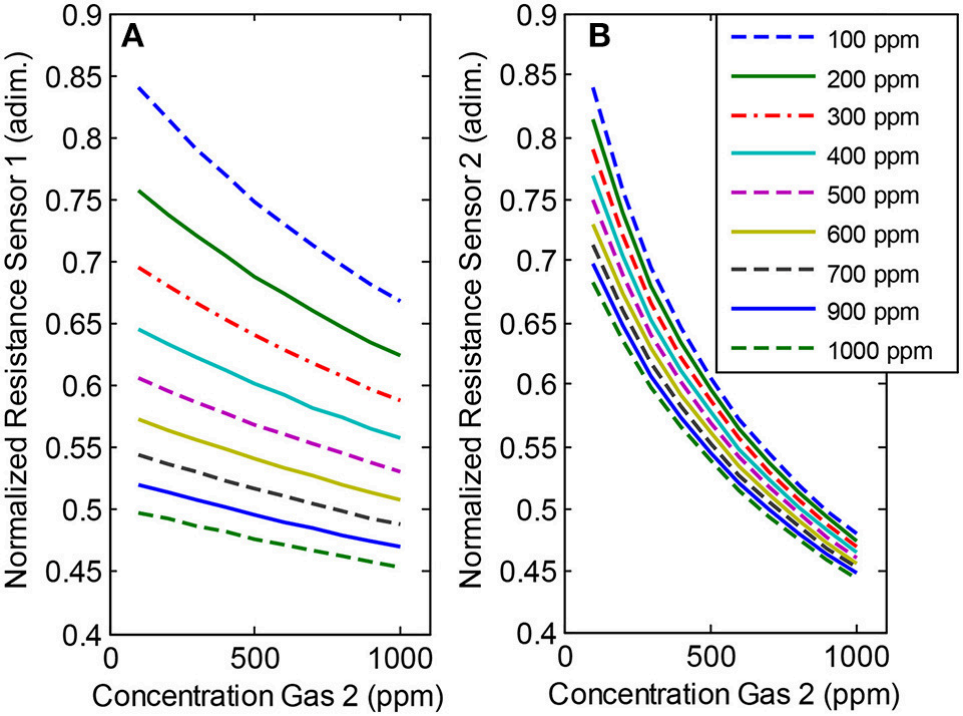
Response of Sensor 1 and Sensor 2 to the binary mixtures of two gases (Gas 1, and Gas 2). The concentration of Gas 1 is represented by the line color (see legend) and the concentration of Gas 2 can be read in the x-axis. Sensor parameters for Sensor 1 and Sensor 2 were, respectively: **(A)** *K*_11_ = *3.3*·*10*^−3^*; K*_12_ = 9.1·*10*^−4^ and **(B)** *K*_21_ = 9.1·*10*^−4^; *K*_22_ = *3.3*·*10*^−3^.

Figure 3 shows an example of the synthetic data for the particular combination of sensor sensitivities: *K*_11_ = *K*_22_ = *3.3*·*10*^−3^*; K*_12_ = *K*_21_ = 9.1·*10*^−4^. We can see the normalized resistance of the sensors along the concentration of first gas (First sensor on the left and second sensor on the right). For each plot, distinct colors and line types represent different concentration levels of the second gas.

### Experimental dataset

We used a portion of the experimental dataset which is described in detail in Burgués et al. (2018). Therefore, only a brief description is given in this section. Two commercial MOX sensors (SB-500-12 and TGS 3870-A04, provided by FIS and Figaro, respectively) were exposed to dynamic mixtures of CO (0–20 ppm) and humid synthetic air (15–70% RH) in a gas chamber. The heater voltage was modulated in the range 0.2–0.9 V, following the manufacturer recommendations. The sensor output was sampled at 3.5 Hz and then interpolated to 100 sample points. We take as sensor output the full response waveform. Consequently, each sensor provides a high dimensional multivariate output (dimension 100), where each sample point is considered a feature.

**Figure 4:**
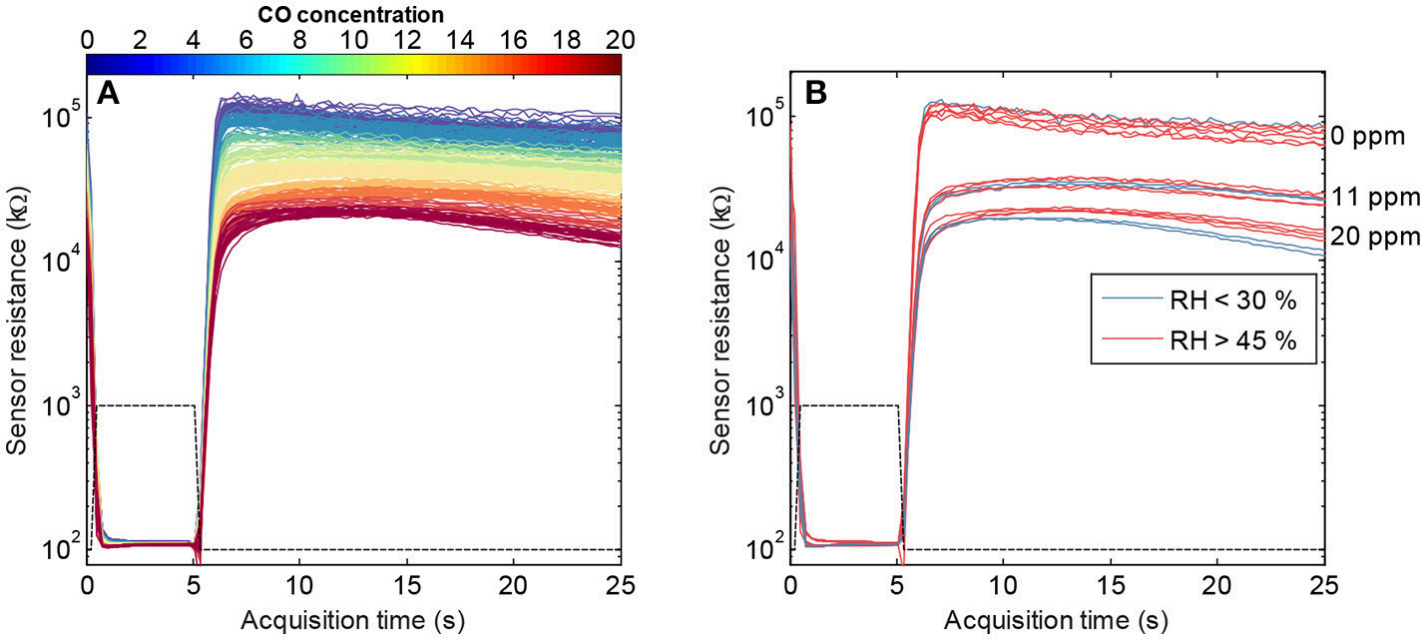
**(A)** Collection of measurements obtained from the SB-500-12 sensor after exposing it to the experimental dataset. Data is represented as a plot of the resistance of the sensor along the acquisition time. The color of the curves represents the concentration of CO. The plot also includes the heater profile applied to the sensor. **(B)** Curves corresponding to concentrations of CO of 0, 11, and 20 ppm, colored by their level of relative humidity (RH). Observe the non-linearities introduced in the pattern due to humidity, and the heteroscedasticity of noise for different concentration levels of CO.

The sensor resistance was measured continuously using a voltage divider with a load resistor of 1 MΩ, once the concentration had reached the steady state in the measurement chamber. Figure 4 shows the logarithmic sensor resistance patterns of the SB-500-12 sensor under different gas conditions. It can be observed that, for certain features, the noise is heteroscedastic because the standard deviation of the sensor response depends on the CO concentration. For example, toward the end of the heating pattern, the variance at 20 ppm is higher than at 11 ppm. Similarly, a non-linear effect of humidity at different concentration levels was found in our dataset: the cross-sensitivity to humidity varied along the heating pattern noise. Therefore, the assumption of some models that the noise has the same standard deviation for each concentration level is unrealistic in our case, and this might lead to a decrease in performance.

### Case studies

#### Case Study I: resolving power against selectivity and noise level

In Case Study I, we aim to analyze how the Resolving Power depends on the *selectivity* (*sel*) of a sensor array. Here we are using the definition of selectivity for an array of non-specific sensors proposed by Johnson and Knapp (2017). Formally, the selectivity of a sensor array is defined as the Cramér-Rao bound analog of the Bayes' rule (Equation 14):

(14) $$\begin{array}{l} sel_{\alpha ,\beta }=\frac{CRB\;(Y_{\alpha }|Y_{\beta })\;\cdot \;CRB\;(Y_{\beta }|Y_{\alpha })}{CRB\;(\text{Y})} \end{array}$$

where ***Y***_α_ and ***Y***_β_ are, respectively, two subsets of chemical stimuli to be distinguished, ***Y*** is the whole stimuli space, and *CRB(**Y**)* is the Cramér-Rao bound analog of the Bayes' rule operator that gives the joint bound of ***Y***_α_ and ***Y***_β_. The reader is referred to Johnson and Knapp (2017) for further details.

According to the previous definition, the *selectivity* of a sensor array measures to what extent the error in the estimation of the concentration is independent of the stimuli. The *selectivity* of a sensor array ranges from 0 to 1. When the sensors of the array are fully specific, the estimation of concentration is independent among stimuli and the selectivity of the sensor array is 1. Conversely, when the sensors of the array become more and more unspecific to the set of mixtures, their responses tend to be undistinguishable and the *selectivity* value of the array decays to 0.

We corrupted the synthetic data generated in section Synthetic Datasets (2001 sensor-pair combinations) with independent Gaussian noise (μ_*i*_ = 0, σ_*i*_ = 0.5, where *i* = 1, 2 is sensor index). Using this data we estimated *V*_*s*_ and *V*_*n*_ for each sensor-pair. Note that, since the dimensionality of the sensor space was two, *V*_*s*_ and *V*_*n*_ were areas. Finally, we computed *N*_*n*_, and *sel* for each sensor-pair.

#### Case Study II: estimation of the resolving power for multivariate response pattern

In Case Study II, we project a highly dimensional sensor space onto lower dimensionality space so as to obtain more accurate estimations of *V*_*s*_, *V*_*n*_, and hence *N*_*n*_. In particular, our sensor space is defined by the concatenation of features of two sensors units (SB-500-12 and TGS 3870-A04) modulated in temperature and exposed to the CO-H_2_O gas mixtures described is section Experimental Dataset. Notice that, due to the temperature profiling we obtained 100 features per sensor (200 features in total), upon which our methodology can be applied.

We used Principal Components Analysis (PCA) projection to reduce the input dimensionality of the sensor space, that is, to create a new set of sensor features (Principal Components, a.k.a. PCs) from the original ones according to their contribution to explain the variance of the dataset. We truncated the PCA model of the data to 2 PCs in order to capture the intrinsic dimensionality of the stimuli space. This criterion is also followed in the rest of sensor spaces generated along the paper (we only create 2-dimensional sensor spaces). If the dimensionality of the stimuli space is not known a priori, the optimum complexity of the PCA model can be estimated by inspecting its plot of eigenvalues against the number of PCs, looking for a “knee” on the line. Once we obtained the new reduced sensor space, we computed its Resolving Power.

#### Case Study III: feature selection based on resolving power

In Case Study III, we use the Resolving Power as a figure of merit to select the most relevant features for gas mixture discrimination from an array of two temperature modulated MOX sensors exposed to CO-H_2_O mixtures.

We constructed three sets of two-dimensional sensor spaces combining selected features from the TGS 3870-A04 and SB-500-12 sensors (from now on TGS and SB sensors). In the first and second sets, the two features came from the same type of sensor (TGS and SB, respectively), whereas in the third set the sensor space was generated joining one feature from each of the sensors. For the sake of simplicity, these sets were called: TGS_1_-TGS_2_, SB_1_-SB_2_, and TGS-SB. Next, we computed the Resolving Power *N*_*n*_ for all the pair-wise combinations of features in a set of sensor spaces.

#### Case Study IV: feature selection based on resolving power: non-linear sensors

In Case Study IV, we present a similar study Case study III. It is similar in the sense that we select relevant features from two MOX for CO-H_2_O mixture discrimination. However, in this case we also consider the effect of non-linearity in sensor responses and the presence heteroscedastic sensor noise for estimating the Resolving Power of the array. The basic idea consists of partitioning the stimuli space in smaller regions so that their corresponding sensors spaces exhibit linearized sensor responses and homoscedastic sensor noise.

Therefore, we divided the stimuli space into four portions according to the midpoints of the concentrations of CO and the RHs, obtaining four gas mixture “subspaces” corresponding to: low concentration of CO and low RH (L-L), low concentration of CO and high RH (L-H), high concentration of CO and low RH (H-L) and high concentration of CO and RH (H-H), respectively. Then, we generated four sets of TGS-SB two-dimensional sensor spaces, one per each for each portion of the stimuli space. Finally, we computed the Resolving Power *N*_*n*_ for all sensor-pair combinations in a set of sensor spaces.

## Results and discussion

### Case Study I: resolving power against selectivity and noise level

**Figure 5:**
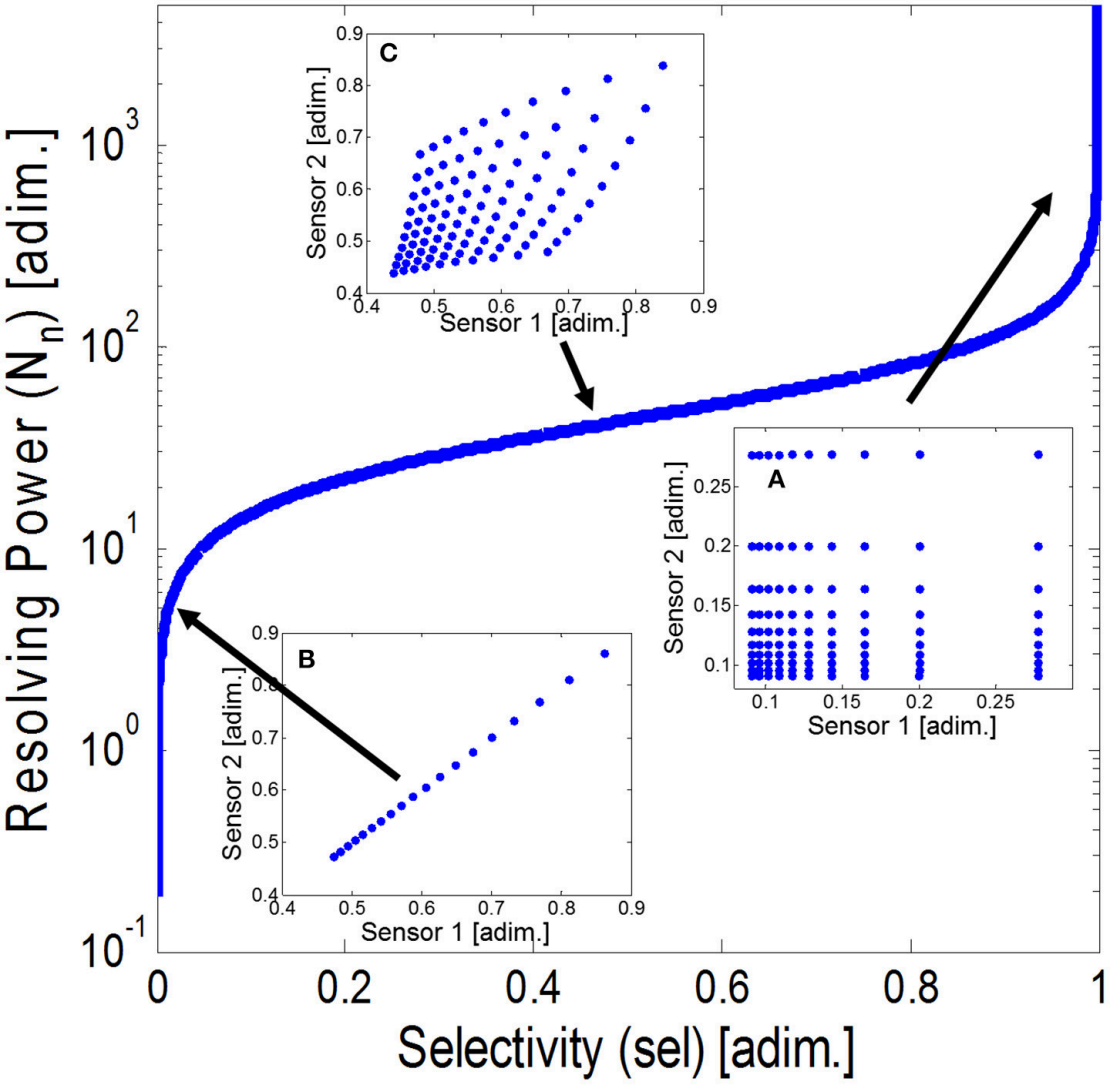
Resolving Power for the set of synthetic sensor-pairs against their selectivity value. The plot includes the sensor space constituted by the sensor pairs for **(A)** totally non-selective sensors, **(B)** partially selective sensors, and **(C)** totally selective sensors. Observe that the area of the sensor space strongly depends on the selectivity of the sensors.

The results of Case Study I are shown in Figure 5, where we represent the Resolving Power against selectivity for all the sensor-pairs. Additionally, we include the sensor space in three particular cases: (a) sensors are totally selective (*sel* = 1), (b) sensors are totally non-selective (*sel* = 0), and (c) sensors are partially selective *(sel* = 0.5).

From the figure, it is evident that when the selectivity tends to 1, the Resolving Power increases dramatically, and on the contrary, when the selectivity tends to 0, the Resolving Power also tends to 0. In the case limits (a) and (b) the responses of the two sensors are, respectively, orthogonal and collinear. These two cases represent the maximum a minimum value of hyper-volume of sensor space. For the intermediate cases, the Resolving Power increases smoothly with the selectivity of the sensor array, and so does the sensor space hyper-volume [as can be seen for case (c)]. Notice that if the Resolving Power is smaller than one no gas mixtures can be distinguished because the volume of the sensor space is smaller than the hyper-volume of noise. If the power of sensor noise was increased, we would obtain a curve with the same shape to the one of Figure 5 but shifted toward lower values of Resolving Power (not shown). Thus, to obtain the same level of Resolving Power in both curves, we would need a higher degree of *selectivity* on the noisier one.

### Case Study II: estimation of the resolving power for multivariate response patterns

**Figure 6:**
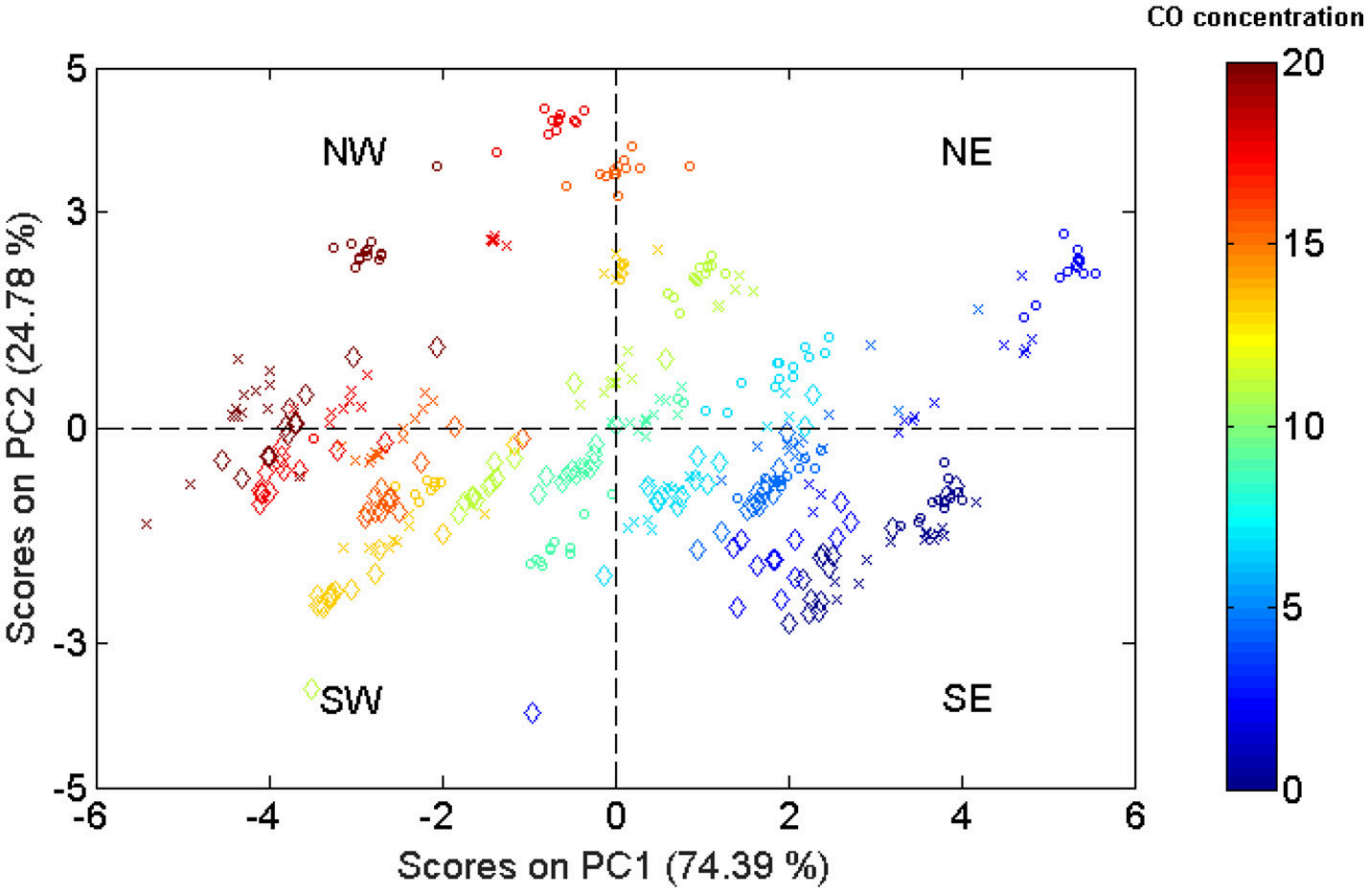
Reduced sensor space obtained from the projection of the original high-dimension sensor space to a 2-dimension space through a PCA projection. Samples were colored according to their concentration of CO, while the marker type represents the level of RH.

The highly dimensional sensor space of Case Study II (200 features) is reduced to a 2-dimensional sensor space by means of PCA projection. The result of this data transformation is shown in Figure 6, where we can see the scores plot of the data samples seen from the new set of sensor features (namely PC1 and PC2). We colored and selected the marker type of the samples according, to respectively, their concentration of CO and RH level. The lower/higher the concentration of CO the bluer/redder the color of the sample. Regarding the RH level, low/medium/high humidity (20–30% RH; 50–55% RH; 65–70% RH) were represented using circle/cross/diamond markers.

Observing the plot, we realize that there are two main directions for the spread of data: South-East to North-West (SE-NW), and South-West to North-East (SW-NE). Both directions have clear chemical meaning: While in SE-NW samples follow the gradient of CO concentration, in SW-NE they follow the gradient of RH level. If we turn now our attention the distribution of samples along these directions, we discover two totally different behaviors: (1) samples are distributed almost linearly and their dispersions seem constant to be independent with respect CO concentration following SE-NW direction, and (2) samples are distributed non-linearly with the RH level, and their dispersions tend to increase to high RHs. We computed the Resolving Power for this reduced sensor space obtaining *N*_*n*_ = 62. From the previous considerations regarding the heteroscedasticity sensor noise, it is acceptable to think that the Resolving Power of this space is underestimated.

### Case Study III: feature selection based on resolving power

**Figure 7:**
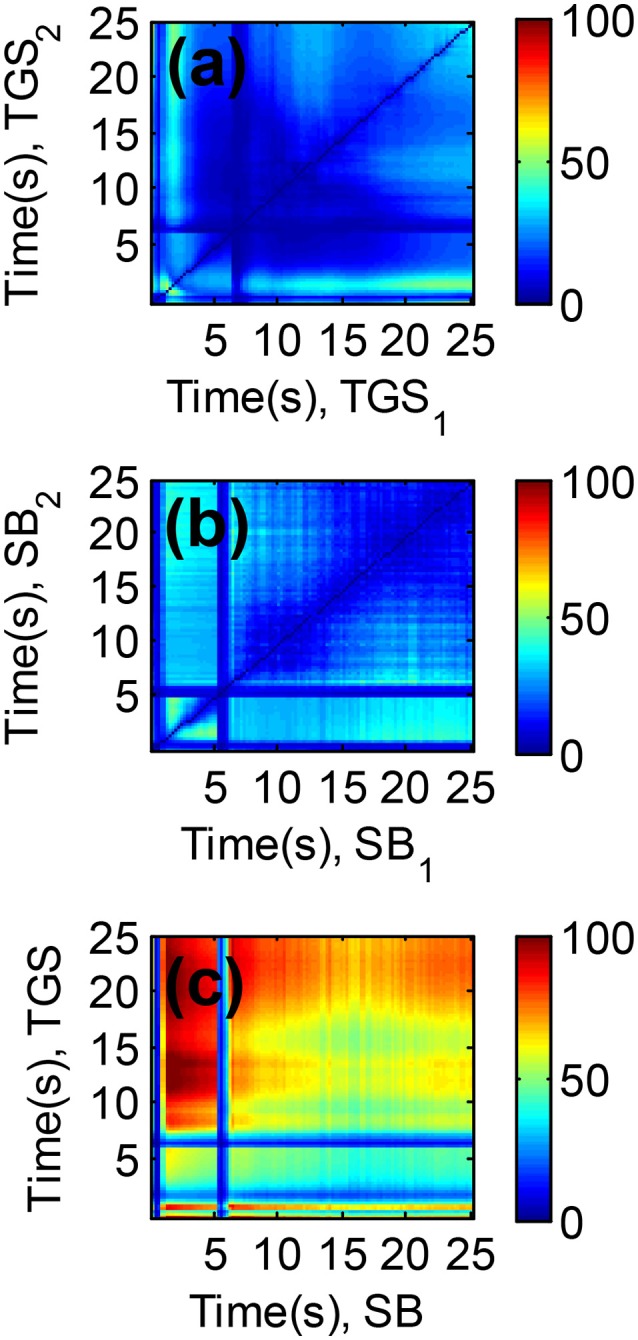
Resolving Power (Nn) obtained for all binary combinations of sensor features from **(a)** the TGS sensor, **(b)** the SB sensor, and **(c)** both sensors. High/Low values of the Resolving Power are colored in red/blue tone, respectively. It is observed an overall increment of *N*_*n*_ in case **(c)** with respect to **(a,b)** due to the combination of features from two different sensor types.

Figures 7a–c shows the color maps for the collection of Resolving Power obtained from each set of sensor spaces: (a) TGS_1_-TGS_2_, (b) SB_1_-SB_2_, and (c) TGS-SB. Colors biased toward red/blue tones denote higher/lower values of the parameter. Note that the distribution of values of *N*_*n*_ is symmetric with respect to the swap of times for Figures 7a,b.

One can confirm similar dependencies between *N*_*n*_ and the features for TGS and SB sensors comparing their distributions in Figures 7a,b. For both sensors, best CO-H_2_O mixture discrimination occurs either combining features obtained at times below 5 s among them (high sensor temperatures) or combining one of these “early” features with another one acquired within the time range that goes from 15 to 25 s (low sensor temperatures). However, none of the sensors achieved Resolving Power above 60 by means of binary combinations of their own features.

When using features of both sensors, we can achieve much higher *N*_*n*_ values, as can be observed in Figure 7c. In particular, the maximum Resolving Power on the figure (*N*_*n*_ = 105) was obtained for the combination of features acquired with the TGS and SB sensors at 12.5 and 1.5 s, respectively. Interestingly, combining features of both sensor types broadens the regions on the colormap were *N*_*n*_ presents high values and variates gracefully. That fact suggests that within these regions the sensors become more specific to the compounds of the mixture, and that they present similar *selectivity* values. Noteworthy, the Resolving Power for the best binary combination of sensor features outperforms the Resolving Power obtained in Case Study II, where we used the full waveform and PCA projection to obtain a new 2-dimensional sensor space.

### Case Study IV: feature selection based on resolving power: non-linear sensors

**Figure 8:**
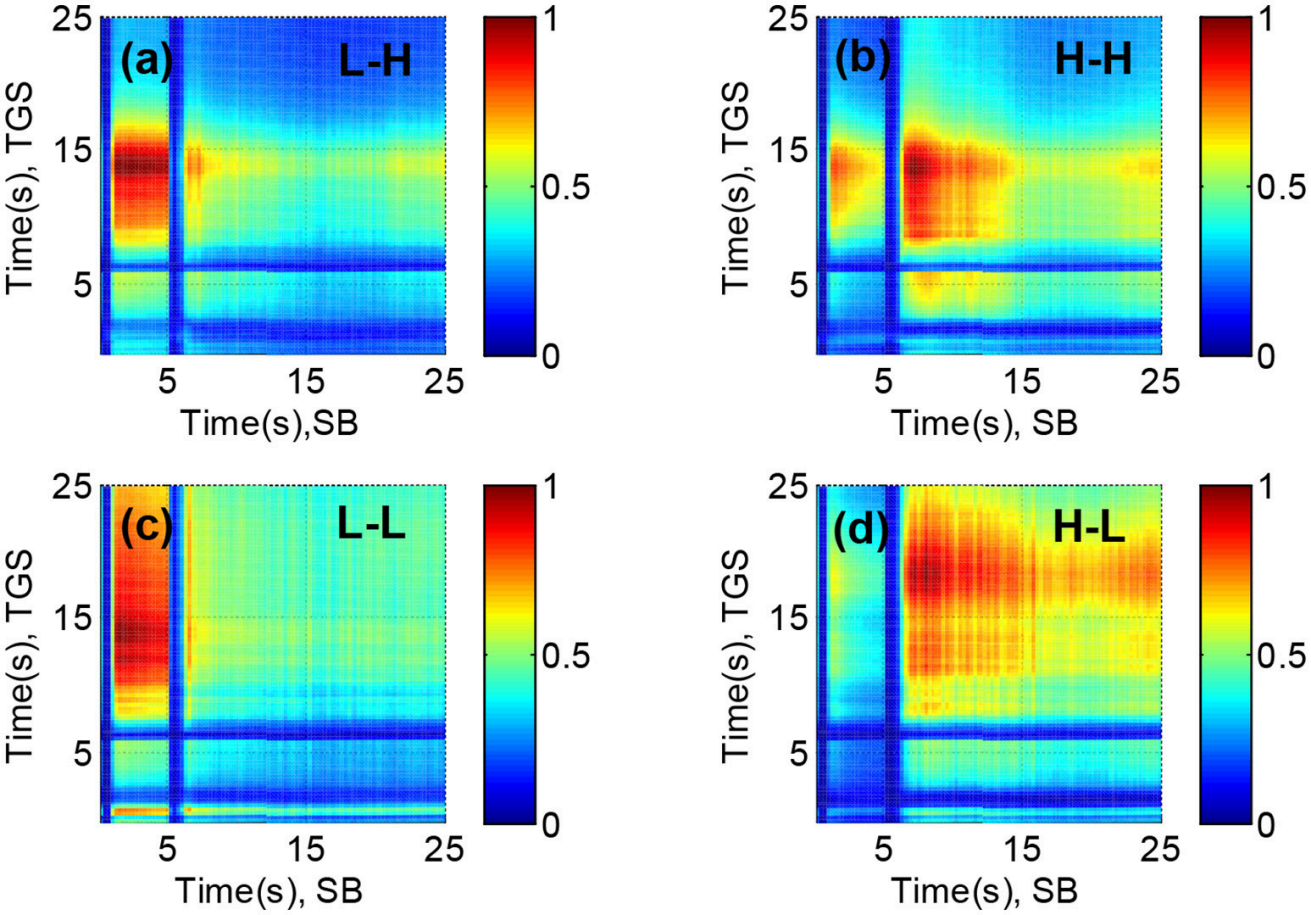
Normalized Resolving Power for all pair-wise combinations of sensor features from a TGS-SB sensor space, and obtained for the partitions of the stimuli space **(a)** L-H, **(b)** H-H, **(c)** L-L, and **(d)** H-L, where L and H denote, respectively, low and high concentrations and their position for each gas type, namely, CO and H_2_O. The Resolving Power is represented using the same color code as Figure 7. The optimum Resolving Power is obtained at different combinations of sensor features for different partitions of the stimuli space.

The Resolving Power of an array of sensor changes for different partitions of the stimuli space when sensors are non-linear (it modifies *V*_*s*_) and sensor noise is heteroscedastic and dependent on gas concentration (it modifies *V*_*n*_). This effect can be appreciated in the colormap plots of Figures 8a–d, where we show the four different collection Resolving Powers obtained from the 2-dimensional TGS-SB sensor spaces, and corresponding the stimuli space partitions: (a) L-L, (b) L-H, (c) H-L, and (d) H-H. To compare the most discriminative sensor-pair combinations among stimuli space partitions, *N*_*n*_ was normalized to its maximum value in each of the colormap plots. The color notation is the same as in Figure 7.

For low concentrations of CO (Figures 8a,c), feature combinations with high *N*_*n*_ tend to concentrate for acquisition times of the SB sensor between 0.5 and 5 s. However, when the concentration of CO increases (Figure 8b), the major part of combinations with high *N*_*n*_ transfer to acquisitions times between 5.5 and 15 s of the same sensor (although the distribution of *N*_*n*_ is not unimodal along the acquisition time). Regarding the RH, low RH levels (Figures 8c,d) obtain a broader scope of features than high RH levels. This behavior is particularly evident for the TGS sensor. The maximal *N*_*n*_ values obtained from the four different stimuli space partitions are 47 (L-L), 21 (L-H), 64 (H-L), and 48 (H-H), respectively. It is worth noting that the sum of the Resolving Power of the four stimuli space partitions (*N*_*n*_ = 180), is much higher than the Resolving Power for the whole stimuli space obtained in Case Study III (*N*_*n*_ = 105). That happens because the combination of features that optimizes the Resolving Power of the sensor array for the whole stimuli space is biased to the discrimination of CO-H_2_O mixtures with low concentrations levels of CO. It may happen too that sensor noise was overestimated for specific concentration ranges.

## Conclusions

In this paper, we have addressed from a practical point of view the relevant problem of finding a figure of merit that characterizes the Resolving Power of a chemical sensor array. The proposed figure of merit is based on the intuitive idea of computing the ratio of the hyper-volume spanned by the sensor signals and that spanned by the noise. Based on this idea, Gardner and Bartlett (1996) and Pearce (Pearce, 2000; Pearce and Sánchez-Montañés, 2004) coined the term *range* of the sensor arrays developing a theoretical framework for the application of this figure of merit to chemical sensor arrays. Since the term range has other meaning in sensor science, we propose to designate this figure of merit as Resolving Power.

Their work was an important advance, but presented significant limitations when applying the Resolving Power to actual sensor array signals. First, the intrinsic dimensionality of the sensor response is limited to the dimensionality of the stimuli space since we have as many independent sources of variance as gases. Even though the dimensionality of sensor/feature space will be higher, we have to compute the hyper-volume with a method that considers only the lower dimensional manifold spanned by the sensor responses. Second, the noise of different types represents new and independent sources of variance with respect to stimuli and makes the sensor responses to move slightly outside the manifold spanned by stimuli. Third, the non-linear nature of chemical sensor responses introduces a high degree of complexity in the transformation from stimuli to sensor space that has not been considered in depth in previous studies (Gardner and Bartlett, 1996; Pearce, 2000; Pearce and Sánchez-Montañés, 2004). Finally, the heteroscedasticity of noise in chemical sensor arrays has not been considered either in previous studies where it has been assumed to be homoscedastic (Gardner and Bartlett, 1996; Pearce, 2000; Pearce and Sánchez-Montañés, 2004).

The methodology proposed in this paper to compute the Resolving Power of the chemical sensor array overcomes these limitations in the following way. First, it finds the hyper-volume spanned by the sensor responses by computing that of its convex hull in sensor space. This captures in a natural way the hyper-volume of the lower dimensional manifold generated by sensor responses. Second, we reduce the dimensionality of the sensor space to match the intrinsic dimensionality of the stimuli space by projecting it to its first principal components. These first two steps of the method allow to successfully computing the Resolving Power of the real chemical sensor array. The fact that the dimensionality reduction is performed before the estimation of the hyper-volume makes it not necessary very intensive computational power. This has been first studied with synthetic data and then with real chemical sensor data. We used datasets with two sensors to illustrate the non-linearity of the sensor responses and the heteroscedasticity of the sensor noise, although our approach can be extended to more complex datasets. Actually, third and fourth limitations of other approaches are addressed by following a stepwise approach dividing the original stimuli space into sub-spaces that provide a more local measure of the Resolving Power. Due to the non-linear response of sensors to gas concentrations and the heteroscedasticity of noise it is preferable to characterize locally the Resolving Power of the chemical sensor array since this will vary with concentration. In Case Study IV, we show the need for a local measure of the Resolving Power since there are differences between the results in the four partitions.

In conclusion, the method proposed is able to successfully compute the Resolving Power of chemical sensor arrays providing a relevant figure of merit that was missing to evaluate these systems.

## Author contributions

LF and JY contributed equally to this work. LF and JY performed the formal analysis and generated the synthetic dataset. JB generated the experimental dataset with actual sensors. LF, JF, AG, and SM defined the methodology. LF, JY, JF, JB, AG, and SM participated in the writing process (preparation of the original draft). LF and JF reviewed and edited the manuscript. LF, AG, and SM conceived the original research.

### Conflict of interest statement

The authors declare that the research was conducted in the absence of any commercial or financial relationships that could be construed as a potential conflict of interest. The reviewer, VP, and handling Editor declared their shared affiliation.
